# Supplementary material for: Patterns of loneliness among high school students: A sociodemographic analysis in Kenya
Source: Glob Ment Health (Camb). 2026 Feb 19;13:e54. doi: 10.1017/gmh.2026.10153 (PMC13112272; doi:10.1017/gmh.2026.10153)
Supplement: Ndetei et al. supplementary material [file S2054425126101538sup001.zip › FINAL Table S2.docx]

Table S2. Multivariable logistic regression of loneliness: adjusted odds ratios (95% CI).

| **term** | **aOR** | **CI_low** | **CI_high** | **p** | **BH-p** |
| --- | --- | --- | --- | --- | --- |
| Gender_Male | 1.033 | 0.740 | 1.440 | 0.850 | 0.859 |
| Gender_Other (Gender) | 3.172 | 0.241 | 41.755 | 0.380 | 0.523 |
| County of School_Makueni | 0.521 | 0.357 | 0.761 | <0.001 | 0.003 |
| Living arrangement_Grandparent(s) | 3.430 | 0.596 | 19.732 | 0.167 | 0.307 |
| Living arrangement_Other (Living arrangement) | 1.391 | 0.036 | 53.534 | 0.859 | 0.859 |
| Living arrangement_Other relative | 2.756 | 0.134 | 56.721 | 0.511 | 0.625 |
| Perceived economic status (3-level)_Not/Not particularly well | 1.267 | 0.913 | 1.758 | 0.157 | 0.307 |
| Perceived economic status (3-level)_Rather/Very well | 0.892 | 0.694 | 1.148 | 0.375 | 0.523 |
| Experiences of Loneliness - How many close friends do you have?_1 friend | 0.485 | 0.283 | 0.832 | 0.009 | 0.023 |
| Experiences of Loneliness - How many close friends do you have?_2 friends | 0.328 | 0.213 | 0.504 | <0.001 | <0.001 |
| Experiences of Loneliness - How many close friends do you have?_3 or more friends | 0.230 | 0.166 | 0.319 | <0.001 | <0.001 |

Notes. Adjusted odds ratios (aOR) with 95% CI from a binomial GLM (logit) with HC3 robust

standard errors. Predictors: sex, urban/rural location, county, living arrangement, perceived

economic status (3 levels), and number of close friends (0/1/2/≥3). One-hot encoding with the

following references: Female, Urban, Nairobi, Two biological parents, Rather/Very well, and ≥3

close friends. Complete-case analysis for the multivariable model. HC3 SEs was used. Grade

was evaluated but excluded due to quasi-separation; bivariate directions unchanged.
