# Supplementary material for: Patterns of loneliness among high school students: A sociodemographic analysis in Kenya
Source: Glob Ment Health (Camb). 2026 Feb 19;13:e54. doi: 10.1017/gmh.2026.10153 (PMC13112272; doi:10.1017/gmh.2026.10153)
Supplement: Ndetei et al. supplementary material [file S2054425126101538sup001.zip › FINAL Figure S1.docx]

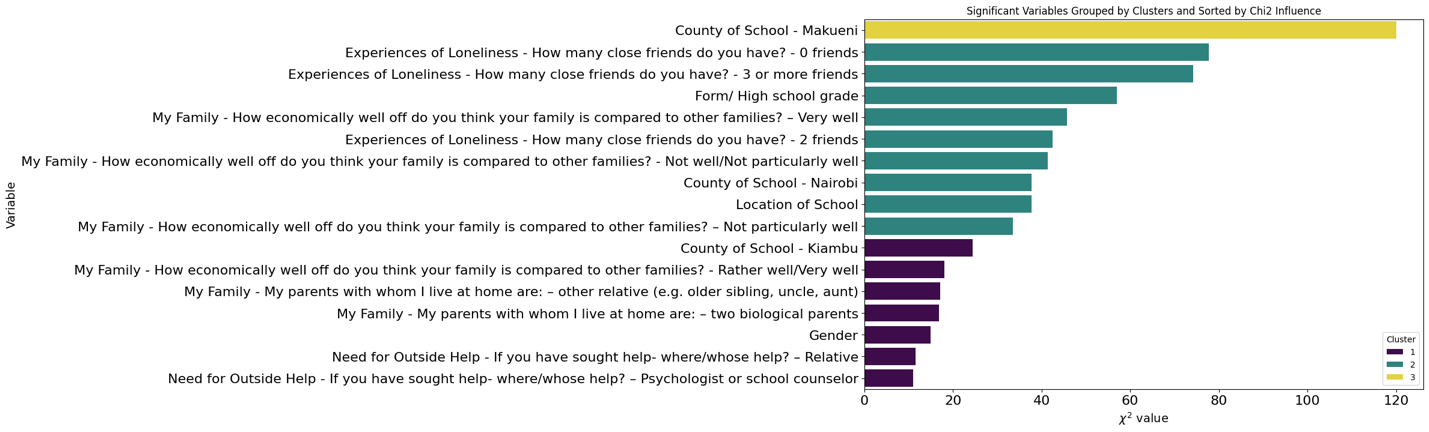


**Figure S1. Assessment of Factors Contributing to Loneliness.** Distance metric: Euclidean on BH-χ²; Ward linkage.

Notes. We conducted a cluster analysis of variables that were found to be significantly associated with loneliness. The cluster analysis was performed taking into account the chi-square values. The elbow method showed a clear elbow at three clusters, suggesting that additional clusters would not significantly improve the partitioning of the data in terms of Within the sum of squares (WSS). This decision was substantiated by a significant drop in distance reduction observed at three clusters, which implies that most variance could be explained within these groups without unnecessary complexity. Dendrogram grouping study factors by the strength of their bivariate association with perceived loneliness. The visualization is provided for descriptive context only; all inferences are based on effect-size estimates (ORs with 95% CIs) and the multivariable model reported in the main text.

The analysis identified several significant factors associated with loneliness, categorized into three distinct clusters based on their chi-square values. *Cluster 1* reported a moderate association and comprised variables with χ² values from 11.000 to 24.394, suggesting their moderate influence on loneliness. It included factors such as demographic influences (county of school Kiambu) and specific familial background with lesser but notable chi-square values. *Cluster 2* showed a high association with levels of loneliness, encompassing variables with χ² values between 33.486 and 45.699. This cluster predominantly included economic status perception and social relational factors (e.g., number of close friends). *Cluster 3* reported a very high association with loneliness and highlights critical areas such as geographical location (county of school Makueni, χ² = 120.089). County-level clustering separated Nairobi, Kiambu and Makueni, with Makueni showing the lowest loneliness prevalence. Lower loneliness in Makueni likely reflects strong extended-family networks typical of this rural county.
