# Supplementary material for: Patterns of loneliness among high school students: A sociodemographic analysis in Kenya
Source: Glob Ment Health (Camb). 2026 Feb 19;13:e54. doi: 10.1017/gmh.2026.10153 (PMC13112272; doi:10.1017/gmh.2026.10153)
Supplement: Ndetei et al. supplementary material [file S2054425126101538sup001.zip › FINAL Table 2B.docx]

Table 2B. Frequency distributions of study factors by loneliness status.

| **Category** | **Factor** | **n (Lonely=0)** | **n (Lonely=1)** | **% Lonely=1** | **% Lonely=0** | **chi2** | **p** | **Cramers V** | **BH-p (factor-level)** |
| --- | --- | --- | --- | --- | --- | --- | --- | --- | --- |
| Female | Gender | 663 | 143 | 17.742 | 80.486 | 1.755 | 0.416 | 0.027 | 0.416 |
| Male | Gender | 1307 | 295 | 18.414 | 80.658 | 1.755 | 0.416 | 0.027 | 0.416 |
| Other | Gender | 3 | 2 | 40.0 | 6.667 | 1.755 | 0.416 | 0.027 | 0.416 |
| Form 1 | Grade | 646 | 139 | 17.707 | 80.478 | 5.467 | 0.141 | 0.048 | 0.164 |
| Form 2 | Grade | 493 | 102 | 17.143 | 80.537 | 5.467 | 0.141 | 0.048 | 0.164 |
| Form 3 | Grade | 563 | 121 | 17.690 | 80.235 | 5.467 | 0.141 | 0.048 | 0.164 |
| Form 4 | Grade | 253 | 75 | 22.866 | 72.107 | 5.467 | 0.141 | 0.048 | 0.164 |
| Rural | Location | 1245 | 236 | 15.935 | 83.170 | 13.201 | <0.001 | 0.075 | <0.001 |
| Urban | Location | 728 | 204 | 21.888 | 76.319 | 13.201 | <0.001 | 0.075 | <0.001 |
| Kiambu | County | 540 | 145 | 21.168 | 76.469 | 37.003 | <0.001 | 0.124 | <0.001 |
| Makueni | County | 705 | 91 | 11.432 | 87.314 | 37.003 | <0.001 | 0.124 | <0.001 |
| Nairobi | County | 728 | 204 | 21.888 | 76.319 | 37.003 | <0.001 | 0.124 | <0.001 |
| 0 friends | Number of friends | 104 | 77 | 42.541 | 46.524 | 100.785 | <0.001 | 0.205 | <0.001 |
| 1 friend | Number of friends | 314 | 95 | 23.227 | 72.647 | 100.785 | <0.001 | 0.205 | <0.001 |
| 2 friends | Number of friends | 323 | 74 | 18.640 | 77.712 | 100.785 | <0.001 | 0.205 | <0.001 |
| 3 or more friends | Number of friends | 1214 | 188 | 13.409 | 85.770 | 100.785 | <0.001 | 0.205 | <0.001 |
| Adoptive parents | Living arrangement | 11 | 3 | 21.429 | 31.048 | 26.633 | <0.001 | 0.107 | <0.001 |
| Biological father | Living arrangement | 49 | 8 | 14.035 | 68.98 | 26.633 | <0.001 | 0.107 | <0.001 |
| Biological mother | Living arrangement | 469 | 122 | 20.643 | 76.679 | 26.633 | <0.001 | 0.107 | <0.001 |
| Foster parents | Living arrangement | 7 | 2 | 22.222 | 22.420 | 26.633 | <0.001 | 0.107 | <0.001 |
| Grandparent(s) | Living arrangement | 43 | 23 | 34.848 | 42.638 | 26.633 | <0.001 | 0.107 | <0.001 |
| Other relative | Living arrangement | 50 | 22 | 30.556 | 48.754 | 26.633 | <0.001 | 0.107 | <0.001 |
| Step-parent + bio | Living arrangement | 56 | 10 | 15.151 | 69.007 | 26.633 | <0.001 | 0.107 | <0.001 |
| Two biological parents | Living arrangement | 1226 | 239 | 16.314 | 82.764 | 26.633 | <0.001 | 0.107 | <0.001 |
| Fairly well | Economic status (3-level) | 621 | 150 | 19.455 | 78.562 | 11.090 | 0.004 | 0.071 | 0.005 |
| Not/Not particularly well | Economic status (3-level) | 284 | 91 | 24.267 | 71.130 | 11.090 | 0.004 | 0.071 | 0.005 |
| Rather/Very well | Economic status (3-level) | 879 | 174 | 16.524 | 82.186 | 11.090 | 0.004 | 0.071 | 0.005 |

Notes. Cross-tabulations of each factor by Lonely (most of the time/always) vs Not lonely, with counts (n), row and column percentages, χ² test of independence, and Cramér’s V (effect size). Benjamini–Hochberg adjustment applied at the factor level. Outcome coded as Lonely=1 for “Most of the time/always”, 0 otherwise. Available-case analysis.
