# Supplementary material for: Patterns of loneliness among high school students: A sociodemographic analysis in Kenya
Source: Glob Ment Health (Camb). 2026 Feb 19;13:e54. doi: 10.1017/gmh.2026.10153 (PMC13112272; doi:10.1017/gmh.2026.10153)
Supplement: Ndetei et al. supplementary material [file S2054425126101538sup001.zip › FINAL Table S1.docx]

Table S1. Bivariate associations with loneliness: prevalence. odds ratios. and effect sizes.

| **Factor** | **Category** | **Reference** | **Prevalence Lonely %** | **n Lonely** | **n Not lonely** | **n Total** | **OR vs ref** | **OR 95% CI lower** | **OR 95% CI upper** | **chi2 (overall)** | **p (overall)** | **Cramers V (overall)** | **BH-p (factor-level)** |
| --- | --- | --- | --- | --- | --- | --- | --- | --- | --- | --- | --- | --- | --- |
| Gender | Female | Female | 17.742 | 143 | 663 | 806 | 1.0 | 1.0 | 1.0 | 1.755 | 0.416 | 0.027 | 0.416 |
| Gender | Male | Female | 18.414 | 295 | 1307 | 1602 | 1.046 | 0.839 | 1.305 | 1.755 | 0.416 | 0.027 | 0.416 |
| Gender | Other | Female | 40.0 | 2 | 3 | 5 | 3.091 | 0.512 | 18.668 | 1.755 | 0.416 | 0.027 | 0.416 |
| Grade | Form 1 | Form 1 | 17.707 | 139 | 646 | 785 | 1.0 | 1.0 | 1.0 | 5.467 | 0.141 | 0.048 | 0.164 |
| Grade | Form 2 | Form 1 | 17.143 | 102 | 493 | 595 | 0.962 | 0.726 | 1.274 | 5.467 | 0.141 | 0.048 | 0.164 |
| Grade | Form 3 | Form 1 | 17.690 | 121 | 563 | 684 | 0.999 | 0.764 | 1.307 | 5.467 | 0.141 | 0.048 | 0.164 |
| Grade | Form 4 | Form 1 | 22.866 | 75 | 253 | 328 | 1.378 | 1.004 | 1.890 | 5.467 | 0.141 | 0.048 | 0.164 |
| Location | Rural | Urban | 15.935 | 236 | 1245 | 1481 | 0.676 | 0.549 | 0.833 | 13.201 | <0.001 | 0.075 | <0.001 |
| Location | Urban | Urban | 21.888 | 204 | 728 | 932 | 1.0 | 1.0 | 1.0 | 13.201 | <0.001 | 0.075 | <0.001 |
| County | Kiambu | Nairobi | 21.168 | 145 | 540 | 685 | 0.958 | 0.754 | 1.218 | 37.003 | <0.001 | 0.124 | <0.001 |
| County | Makueni | Nairobi | 11.432 | 91 | 705 | 796 | 0.461 | 0.352 | 0.602 | 37.003 | <0.001 | 0.124 | <0.001 |
| County | Nairobi | Nairobi | 21.888 | 204 | 728 | 932 | 1.0 | 1.0 | 1.0 | 37.003 | <0.001 | 0.124 | <0.001 |
| Number of friends | 0 friends | 3 or more friends | 42.541 | 77 | 104 | 181 | 4.781 | 3.429 | 6.666 | 100.785 | <0.001 | 0.205 | <0.001 |
| Number of friends | 1 friend | 3 or more friends | 23.227 | 95 | 314 | 409 | 1.954 | 1.482 | 2.575 | 100.785 | <0.001 | 0.205 | <0.001 |
| Number of friends | 2 friends | 3 or more friends | 18.639 | 74 | 323 | 397 | 1.479 | 1.101 | 1.988 | 100.785 | <0.001 | 0.205 | <0.001 |
| Number of friends | 3 or more friends | 3 or more friends | 13.409 | 188 | 1214 | 1402 | 1.0 | 1.0 | 1.0 | 100.785 | <0.001 | 0.205 | <0.001 |
| Living arrangement | Adoptive parents | Two biological parents | 21.429 | 3 | 11 | 14 | 1.399 | 0.387 | 5.052 | 26.633 | <0.001 | 0.107 | <0.001 |
| Living arrangement | Biological father | Two biological parents | 14.035 | 8 | 49 | 57 | 0.838 | 0.392 | 1.791 | 26.633 | <0.001 | 0.107 | <0.001 |
| Living arrangement | Biological mother | Two biological parents | 20.643 | 122 | 469 | 591 | 1.334 | 1.047 | 1.701 | 26.633 | <0.001 | 0.107 | <0.001 |
| Living arrangement | Foster parents | Two biological parents | 22.222 | 2 | 7 | 9 | 1.466 | 0.303 | 7.098 | 26.633 | <0.001 | 0.107 | <0.001 |
| Living arrangement | Grandparent(s) | Two biological parents | 34.848 | 23 | 43 | 66 | 2.744 | 1.623 | 4.638 | 26.633 | <0.001 | 0.107 | <0.001 |
| Living arrangement | Other relative | Two biological parents | 30.556 | 22 | 50 | 72 | 2.257 | 1.342 | 3.797 | 26.633 | <0.001 | 0.107 | <0.001 |
| Living arrangement | Step-parent + bio | Two biological parents | 15.151 | 10 | 56 | 66 | 0.916 | 0.461 | 1.821 | 26.633 | <0.001 | 0.107 | <0.001 |
| Living arrangement | Two biological parents | Two biological parents | 16.314 | 239 | 1226 | 1465 | 1.0 | 1.0 | 1.0 | 26.633 | <0.001 | 0.107 | <0.001 |
| Economic status (3-level) | Fairly well | Rather/Very well | 19.455 | 150 | 621 | 771 | 1.220 | 0.959 | 1.553 | 11.090 | 0.004 | 0.071 | 0.005 |
| Economic status (3-level) | Not/Not particularly well | Rather/Very well | 24.267 | 91 | 284 | 375 | 1.619 | 1.215 | 2.156 | 11.090 | 0.004 | 0.071 | 0.005 |
| Economic status (3-level) | Rather/Very well | Rather/Very well | 16.524 | 174 | 879 | 1053 | 1.0 | 1.0 | 1.0 | 11.090 | 0.004 | 0.071 | 0.005 |

Notes. For each factor category: prevalence of loneliness (%), counts (n), odds ratio (OR) vs the stated reference with 95% CI; plus overall χ² and Cramér’s V for the factor. Benjamini–Hochberg–adjusted p-values reported at the factor level. Reference categories used. Female (sex), Urban (location), Nairobi (county), ≥3 close friends (number of friends), Two biological parents (living arrangement), Rather/Very well (perceived economic status). Lonely defined as above; available-case analysis.
